# What is effective classroom dialog? A comparative study of classroom dialog in Chinese expert and novice mathematics teachers’ classrooms

**DOI:** 10.3389/fpsyg.2022.964967

**Published:** 2022-09-23

**Authors:** Wenjun Zhao, Jing Ma, Yiming Cao

**Affiliations:** ^1^School of Mathematical Sciences, Sichuan Normal Univeristy, Chengdu, China; ^2^Research Center for Mathematics, Beijing Normal University at Zhuhai, Zhuhai, China, Laboratory of Mathematics and Complex Systems (Ministry of Education), School of Mathematical Sciences, Beijing Normal University, Beijing, China; ^3^Faculty of Education, International Center for Research in Mathematics Education, Beijing Normal University, Beijing, China

**Keywords:** effective classroom dialogue, comparative study, Chinese mathematics classrooms, expert teachers, novice teachers

## Abstract

Conducting effective classroom dialog is an important foundation for high-quality classrooms. This study investigates the characteristics of effective classroom dialog from the perspective of Chinese mathematics classrooms. Classroom videotapes from 40 expert and 33 novice teachers were selected from a national project and analyzed through a developed coding framework. Results showed that the dominant types of dialog in expert teachers’ classrooms were related to Basic Knowledge, Construction, Analysis, and Personal Information. Compared to novice teachers, expert teachers’ classrooms have a significantly lower proportion of dialog on Basic Knowledge and significantly higher proportions of dialog on Personal Information and Speculation. Based on expert teachers’ classrooms, the characteristics of effective classroom dialog in the Chinese context were discussed. The analytical framework for classroom dialog developed in this study could be a powerful tool for subsequent research. Suggestions are provided on increasing the effectiveness of classroom dialog.

## Introduction

It has been widely documented that effective classroom dialog can promote students’ learning outcomes by facilitating their robust learning, deepening their understanding of knowledge, and developing their critical thinking skills (Mercer and Dawes, 2014; Resnick et al., 2015; van der Veen et al., 2017; Howe et al., 2019). Thus, the characteristics of effective classroom dialog and strategies to facilitate effective classroom dialog are of central interest to researchers (Howe and Abedin, 2013; Song et al., 2019).

While studies on classroom dialog have grown rapidly in the past 20 years, most have been conducted in Western contexts (Song et al., 2019). This study argues that viewing the characteristics of effective classroom dialog from the perspective of Chinese mathematics classrooms is necessary and valuable. Learning about teaching and learning from high-achieving education systems has become a trend (Li and Shimizu, 2009), and Chinese students constantly outperform their Western counterparts in mathematics in international comparative studies, such as the TIMSS (Trends in International Mathematics and Science Study) and PISA (Programme for International Student Assessment). It is widely agreed that such outstanding performances are related to the high-quality of instruction in Chinese classrooms (Stigler and Hiebert, 2009; Fan et al., 2015). By focusing on classroom dialog, an important foundation for high-quality instruction, this study investigates the wisdom of Chinese mathematics teaching.

The beginning of the 21st century has seen a wave of international comparative studies of classroom teaching and learning, with TIMSS video study and the LPS (The Learners’ Perspective Study) being the main representatives. Many conclusions regarding the characteristics of Chinese mathematics classrooms have been drawn during this time (Leung, 2005; Huang, 2006). However, classroom teaching and learning have greatly changed in the last two decades as China’s current mathematics curriculum reform has advanced (Cao and Leung, 2018). There is a need to examine what constitutes an effective classroom in the reform context. In general, China’s current mathematics curriculum reform calls for a transition from examination-oriented to quality-oriented instruction to cultivate students as lifelong learners with problem-posing and solving abilities, improved communication and collaboration skills, and greater creativity (Cao and Leung, 2018). In terms of classroom instruction, it aims to shift from a teacher-centered approach (e.g., teacher control, lecturing, rote memorization, and extensive exercises and practices), to a more student-centered approach (e.g., self-regulated learning, exploratory and hands-on activities, group discussion, and project work) (Ministry of Education, 2001, 2011). Another important and recent trend in China’s current curriculum reform is the emphasis on cultivating students’ high-order thinking abilities (Ministry of Education, 2011, 2022). China’s “Core Literacy for 21st Century Student Development” (Lin, 2016) and “5C Model for 21st Century Core Literacy” (Wei et al., 2020) consider high-order thinking abilities—including critical, creative, innovative, and problem-solving thinking—as key to core literacy development. These curriculum reform ideas and initiatives will be considered when constructing the analytic framework and discussing effective classroom dialog.

Furthermore, this study adopts an expert-novice comparison design, often used by cognitive psychologists to study knowledge in specialized domains (Berliner, 2001). Expert teachers are those with high attainments in classroom teaching, student achievement, and research (Goodwyn, 2016). Their classroom teaching practices are models and motivators for other teachers. A comparison with novice teachers can better reveal the unique characteristics of expert teachers’ classrooms and provide suggestions for improving novice teachers’ classroom practices.

The identification and selection of expert teachers vary from study to study (Brandt, 1986; Leinhardt, 1986), making comparison between studies difficult. There have been growing calls to identify expert teachers using a systematic and rigorous teacher credential mechanism (Berliner, 2001). China’s nationwide professional title system for primary and secondary school teachers—which is based on teachers’ educational background, teaching performance, student achievement, and teaching and research ability (Gao, 2016)—helps this study identify teachers who enjoy a good reputation in their teaching field and are recognized as experts in education and teaching.

Based on the above background and purpose, this study addresses the following questions:

1. What are the characteristics of Chinese expert mathematics teachers’ classroom dialog?
2. What are the similarities and differences between Chinese expert and novice mathematics teachers’ classroom dialog characteristics?

The first question explores what constitutes effective classroom dialog in Chinese expert teachers’ classrooms. The second question explores the similarities and differences between expert and novice teachers to examine the former’s classrooms’ characteristics and provide suggestions for improving the latter’s competencies for conducting effective classroom dialog.

## Literature review

### Notions and characteristics of effective classroom dialog

The existing literature includes various definitions for classroom dialog, all highlighting interaction as a key feature (Mercer et al., 2019). This study adopts Howe and Abedin’s (2013) definition of classroom dialog, i.e., communication where “one individual addresses another individual or individuals and at least one addressed individual replies” (Howe and Abedin, 2013, p. 326), as it is broad enough to encapsulate other definitions’ many commonalities (Song et al., 2019).

Much of the research on classroom dialog builds on Vygotsky (1978) socio-cultural theory, which discusses the relationships between thought, action, communication, and culture (Alexander, 2015). The central view of socio-cultural theory emphasizes that society can be seen as a network of shared activity systems whose interactions are mediated by language, rules, community, and division of labor (Lantolf, 2000). Learning is an activity in which the subject constructs meaning through dialogical interaction in a socio-cultural context. In learning activities, language is the medium, subjective meaning construction is the core, and cultural context is the foundation.

It has been widely documented that effective classroom dialog helps students exchange different ideas, develop critical thinking, and strengthen their understanding of knowledge, resulting in improved learning outcomes (Mercer and Dawes, 2014; van der Veen et al., 2017; Howe et al., 2019). As such, researchers, policymakers, and educators are interested in learning how to facilitate effective classroom dialog (Zuiker and Anderson, 2021).

Researchers use “scaffolding “as a metaphor for effective classroom dialog and describe how teachers use dialog to build channels that guide students’ independent inquiry, develop analytical and problem-solving skills, and promote critical thinking through discussion, questioning, and reflection, resulting in transferability and innovative capabilities (Bakker et al., 2015). Alexander (2008) proposed that dialogic teaching should be collective, reciprocal, supportive, cumulative, and purposeful. Liu (2013) echoed this, claiming that the ideal state of teacher-student dialog is one in which students express their mathematical ideas freely and openly. When teachers listen and respond effectively to their students’ ideas, both parties can develop an understanding and create meaning in a discourse community. Howe et al. (2019) identified several productive forms of classroom dialog, including open questions, elaboration and reasoning, coordination across contributions, and metacognition.

### Frameworks regarding the analysis of classroom dialog

Classroom dialog can be analyzed using a quantitative or qualitative approach. Each approach has its strengths and weaknesses, which makes them suitable for different purposes. A quantitative method often uses a coding scheme to analyze classroom videotapes/transcripts and look for patterns or relationships. For instance, many existing studies have adopted the FIAS to code classroom activities every 3 s to determine the teaching style based on the frequency distribution and interaction of each code (Martina et al., 2021; Zhao and Boonyaprakob, 2022). Qualitative methods often focus on certain classroom moments and seek the deeper meaning behind the dialog. A qualitative analysis framework should be open and sustainable to account for classroom dialog’s social and cognitive nature and probe how language influences thinking and knowledge construction.

With both quantitative and qualitative methods, the unit of coding can vary from a single word to a sentence to several sentences, as long as it is well defined (Chin, 2006; Wells and Arauz, 2006). Howe et al. (2019) highlighted two frequently adopted macro-and micro-level classroom dialog analysis methods: turn-level and lesson-level, respectively. Turn-level analysis refers to the coding conducted at each turn (identified *via* speaker switch). A turn can be coded by more than one code if applicable. Lesson-level analysis can be applied if certain aspects of the lesson, such as the classroom atmosphere, are difficult to describe in a micro manner. In such a case, the classroom dialog can be rated comprehensively and holistically.

Several frameworks are available to analyze classroom dialog (Song et al., 2020), the most well-known being IRF (Sinclair and Coulthard, 1975), FIAS (Flanders, 1970), and CLASS (Pianta et al., 2008). However, these frameworks mainly focus on the form of classroom dialog. For instance, IRF categorizes classroom dialog by initiative, response, feedback, or evaluation, while FIAS lists 10 kinds of teacher or student behaviors. None can reflect the connotation and quality of the dialog. Therefore, some research teams have set out to develop frameworks that focus more on the effectiveness of classroom dialog (Howe et al., 2019). For instance, the Cambridge Educational Dialogue Research Group (see Howe et al., 2019) proposed a coding scheme to represent productive dialog that includes items like elaboration, reasoning, coordination, agreement, querying, reference back, and reference widely. Song et al. (2020) reviewed coding frameworks for classroom dialog over the past two decades, concluding that a dialogic framework should encapsulate six themes: prior knowledge, personal information, analysis, generalization, speculation, and uptakes.

However, these analytical frameworks in the literature have different indicators and standards, making it difficult to compare findings across studies. Given the rapid growth of research in this field, a comprehensive, general, and scientific analytical framework for classroom dialog is greatly needed to make cross-study comparisons more applicable and effective.

### Research on classroom dialog in Chinese mathematics classroom

A systematic investigation of Chinese mathematics classrooms was conducted at the beginning of the 21st century through several international comparative studies of classroom teaching and learning, such as TIMSS video study and LPS.

The TIMSS 1999 video study revealed that classroom dialog in the classrooms of Hong Kong SAR is characterized by (1) whole-class interaction; (2) teacher-led lectures; (3) more reasoning and argumentation and more fully-developed expression; (4) a more coherent classroom; and (5) a higher likelihood of student engagement in mathematics learning, etc (Leung, 2005).

Four regions in China (Hong Kong SAR, Macau SAR, Shanghai, and Beijing) participated in the LPS Study, which recorded 10–15 consecutive lessons from at least three representative Grade 8 mathematics teachers in each region. The results showed that in Chinese classrooms, whole-class interaction was the main type of interaction behavior in Chinese mathematics classrooms; the teacher initiated most interactions, and the average volume of teacher discourse was 6.6 times that of students (Cao and He, 2009).

Huang (2006) synthesized the literature on research on teaching and learning in Chinese mathematics classrooms to summarize the characteristics of Chinese mathematics classrooms. Those related to classroom dialog included emphasizing on explanation and illustration, mathematical reasoning, development and construction of knowledge, and procedural problem practice and emphasizing on format, mathematical connections, stimulating questioning and teacher-student interaction, and a lack of realistic contextual connections.

An expert-novice comparison design was often adopted when discussing classroom teaching and learning effectiveness. Generally, expert teachers gave students more opportunities to express their ideas, be more sensitive to classroom tasks, and be more perceptive of social situations while problem-solving (Wang and Ye, 2020). In addition, expert teachers paid greater attention to developing students’ mathematical and high-order thinking abilities (Huang and Li, 2012); gave higher-quality explanations that were more accurate and critical (Song et al., 2021a); shared better knowledge regarding eliciting and responding to student thinking; and encouraged students to generate their ideas and work (Gai et al., 2009). In contrast, novice teachers tended toward “teacher-centred interactive dialog,” characterized by “teacher’s question, student’s answer, and teacher’s evaluation.” (Gai et al., 2009, p.38). Novice teachers often repeated students’ answers and offered few opportunities for student participation (Wang and Ye, 2020).

The past two decades of curriculum reform have wrought many changes in classroom teaching and learning. However, few recent studies have investigated classrooms; the few local studies that were conducted mostly featured small sample size and yielded hard-to-generalize findings.

## Methodology

### Research context and participants

According to the latest standard for China’s teachers’ professional title promotion system released on August 28, 2015 (Ministry of Education, 2015), primary and secondary school teachers can be assessed as level 3, 2, 1, senior, or exceptional. This study defines novice teachers as those having fewer than 5 years of teaching experience and a level 3 or 2 professional title, and expert teachers as those with a senior or exceptional title.

According to the Ministry of Education (2015), senior and exceptional level teachers are required to (1) work and teach on the front line of education for a long time, act as guides and mentors to promote young students’ healthy growth, excel at classroom teaching and counseling tasks, and achieve outstanding teaching and education results; (2) have an in-depth and systematic mastery of the curriculum, professional knowledge of the subjects they teach, outstanding educational and teaching performance, exquisite teaching art, and a unique teaching style; (3) lead and direct educational and teaching research, achieve creative results in educational thinking, curriculum reform, teaching methods, etc., apply them widely in their teaching practice, and play an exemplary and leading role in implementing quality education; and (4) have a bachelor’s degree or higher.

Level 2 and 3 teachers are required to (1) be relatively proficient in the principles and methods of educating students and perform the work of classroom teachers and counsellors with good educational results; (2) show mastery of basic pedagogical and psychological theories and knowledge, have the necessary professional knowledge for the subjects they teach, independently master the syllabus and teaching materials of the subjects they teach, correctly impart knowledge and skills, and have good teaching effectiveness; (3) master educational and teaching research methods, actively conduct educational and teaching research, and practice innovation; and (4) either have a master’s degree; or have a bachelor’s degree, complete a 1-year apprenticeship, and pass the examination; or have a college degree and have taught in primary and junior high school for more than 2 years; or have a secondary teacher training school degree and have taught in elementary school for more than 3 years (Ministry of Education, 2015).

The lessons analyzed in this study were selected from a larger project that has systematically collected more than 500 junior secondary level mathematics lessons from over 10 provinces in Mainland China. Lesson selected for analysis in this study had to: (1) have been videotaped in the past 5 years (2017–2021); (2) be a regular lessons (not a public or competition lessons); and (3) cover as many provinces and districts as possible. Additionally, each school had to select one teacher and one lesson from that teacher; if a teacher had multiple lessons, their second or third was selected to reduce video disruptions. This process yielded lessons from 40 expert (averaging 22 years of teaching experience) and 33 novice teachers (averaging 3 years of teaching experience) in various domains (40 Algebra lessons; 20 Geometry lessons; three Statistics lesson; and 10 inquiry-based lessons). The project received ethical approval from Beijing Normal University in January 2017 and all participants, including principles, teachers, students, and guardians, signed informed consent forms before data collection.

### Analytical framework

The analytical framework of this study was adapted from the coding instrument for productive classroom dialog developed by Song et al. (2021b), which contains nine categories: prior knowledge, personal information, analysis, coordination, speculation, construction, agreement, challenge, and instruction /guide.

Song et al. (2021b) framework is based on a systematic review of frameworks for coding toward classroom dialog over the past 30 years and many years of research experience. The framework is suitable for examining the effectiveness of classroom dialog as it not only focuses on dialog form but also reflects its function and quality.

In addition, this study developed sub-categories for the nine categories proposed by Song et al. (2019) to better reflect the characteristics of the mathematics subjects and align with the current curriculum reform in China, using the process described below.

First, the relevant literature was analyzed to look for potential categories. Using word frequency analysis, the research team analyzed recent policy documents regarding the development of future students (e.g., OECD future of education and skills 2030; PISA 2021/2018/2012 mathematics framework; develop students’ core competency in China), the latest mathematics curriculum standards from China, the United States, the United Kingdom, Singapore, and Australia, and 180 papers related to classroom dialog in mathematics classes rooms to examine figure student development goals from local and international perspectives and capture the specific characteristics of classroom dialog in mathematics.

Second, we identified 30 high-frequency keywords to provide practical and valuable references for the development of the framework. Through extensive discussion, two experts in mathematics education further sorted these high-frequency keywords into 10 key student development objectives—basic knowledge, problem-solving, logical reasoning, practical exploration, cooperative communication, mathematical expression, transfer application, critical innovation, interest, and literacy—to help develop sub-categories for the study’s analytical framework.

Third, referencing the above keywords, the research team developed two to four sub-categories for each category. In addition, the research team invited 20 experts in mathematics education to evaluate the framework and then revised it based on their feedback. After the revision, all 20 experts agreed that the framework had good content validity.

Lastly, three researchers majoring in mathematics education coded 15 lessons separately. The coding results were compared and any differences were resolved through extensive discussion. Descriptions of the coding framework were revised to reduce confusion. Using the final framework, three researchers coded 10 lessons separately, and the inter-rater reliability is 91% (number of dialog turns with consistent codes divided by the total number of dialog turns).

The final framework is presented in Appendix 1.

### Analytical process

All the lessons were transcribed verbatim, and the transcripts were then analyzed. Classroom dialog was coded at the turn-level, i.e., a contribution to an exchange made by a participant in a single speaking turn or its constituents. Multiple codes could be applied to each turn of dialog, but multiple instances of a single code within one turn were marked only once. An example of the coding result is presented in Appendix 2.

Three researchers coded the lessons, and the coding results were double-checked. In the coding process, the researchers would constantly refer back to the classroom videotapes to ensure a good understanding of dialog context and improve the coding accuracy. The whole coding process was conducted using the Classroom Teaching Analysis Platform^1^ developed by the research team.

In the end, the proportion of each code in expert and novice teachers’ lessons was calculated and compared. We chose proportion rather than frequency because generally, dialog turns (the analytical unit) in expert teachers’ classrooms were much fewer than those in novice teachers’ classrooms. As such, the proportion could better reflect the distribution of different types of classroom dialog. In addition, a *t*-test (two-tail) was conducted (*Via* SPSS) to examine the significance level of the differences between expert and novice teachers.

## Results

This study’s results are reported in two parts. The first part presents the characteristics of classroom dialog by expert teachers to reveal the characteristics of effective classroom dialog in China. The second part compares and contrasts the characteristics of expert and novice teachers’ classroom dialog.

### Characteristics of classroom dialog in expert teachers’ classrooms

**Figure 1:**
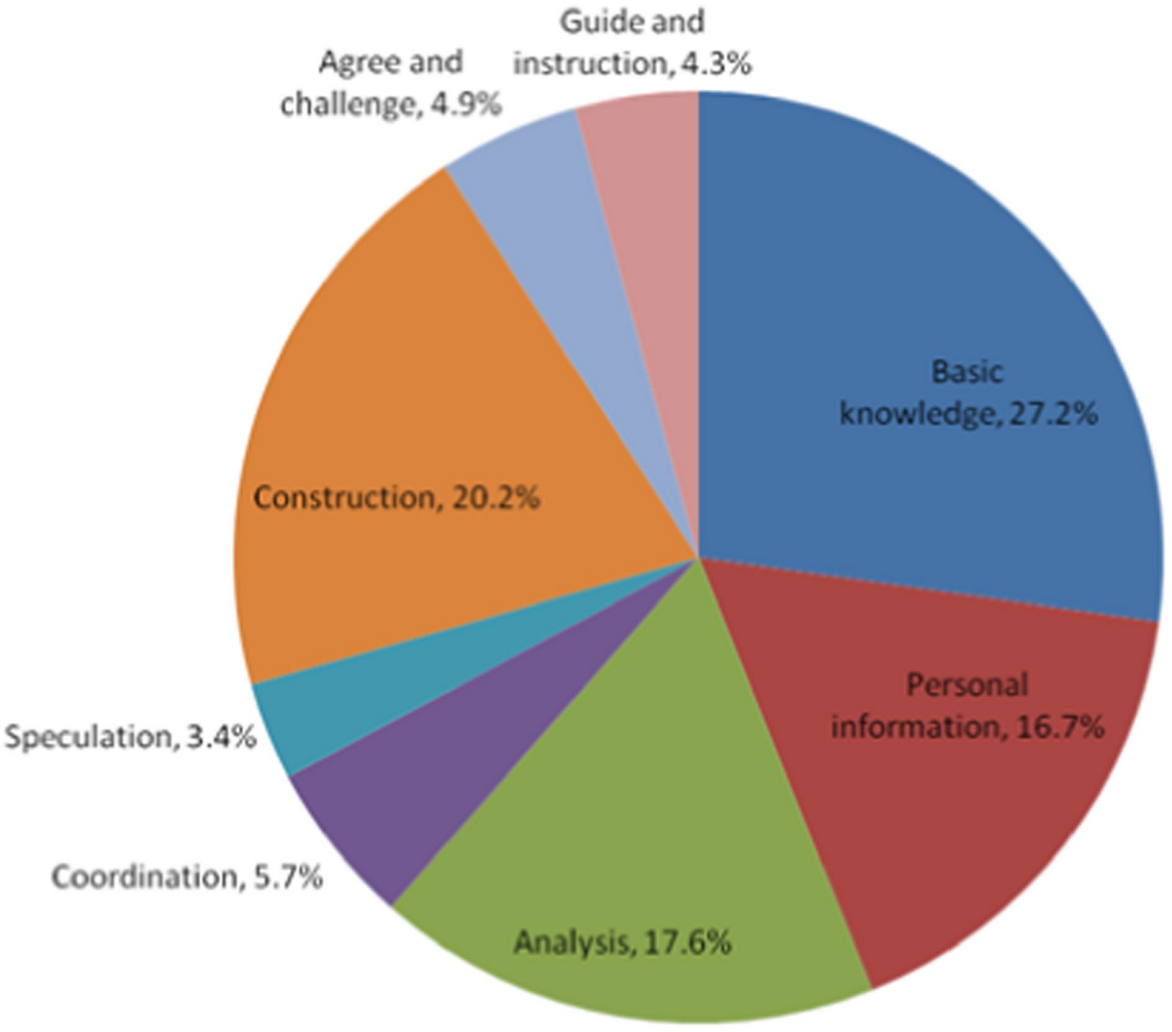
Distribution of dialog types in expert teachers’ classrooms (first-level codes).

From Figure 1, we can see that the proportion of various classroom dialog types from largest to smallest is Basic Knowledge (27.2%), Construction (20.2%), Analysis (17.6%), Personal Information (16.7%), Coordination (5.7%), Agree and challenge (4.9%), Guide and instruction (4.3%), and Speculation (3.4%).

**Figure 2:**
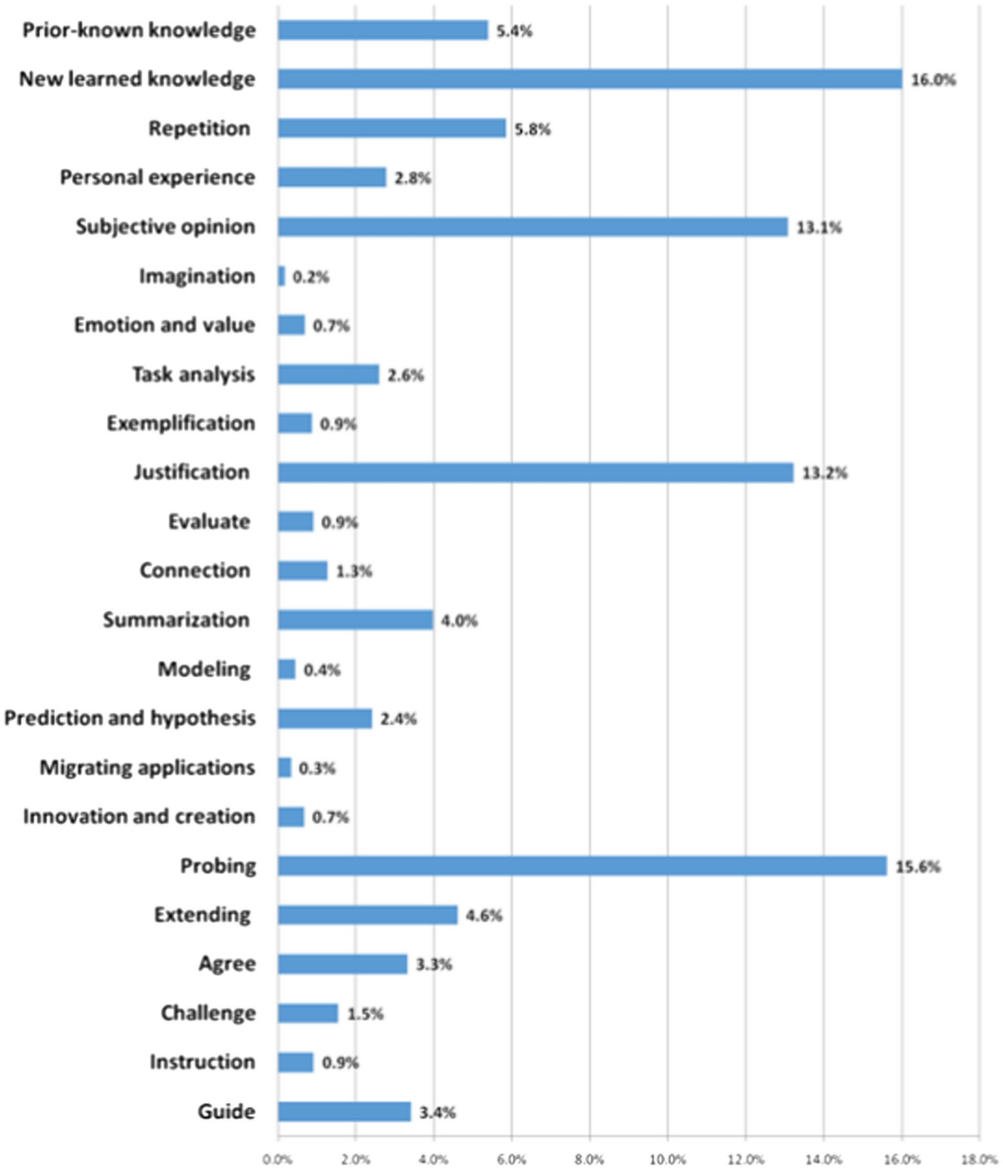
Distribution of dialog types in expert teachers’ classrooms (second-level codes).

Basic Knowledge contains mathematical concepts and symbols, relationships and operations, the history of mathematics, general knowledge, and other knowledge. Typically, classroom dialog related to basic knowledge accounts for the largest proportion, as learning basic knowledge is the basic task of mathematics classrooms. From Figure 2, we can see that in addition to a big proportion of dialog on newly learned knowledge (16%), there was also some dialog on prior-known knowledge (5.4%). By reviewing prior-known knowledge, teachers can deepen students’ memory and lay the foundation for new knowledge. Leinhardt (1989) similarly reported that expert teachers often “used something familiar to teach something new” (p.66). In addition, expert teachers often repeated students’ answers to emphasize relevant knowledge and deepen students’ memory (5.8%).

The proportion of the code “Construction” (including the two sub-codes, Probing, and Extending) ranked second. From Figure 2, we can see that most of the dialog related to Construction was Probing (15.6% out of 20.2%), referring to the dialog in which the teacher/student builds on prior utterances to dig deeper. For instance, “How did you come up with this idea?” and “You said that the two triangles are similar; can you explain why?” The results showed that expert teachers often advanced their students’ thinking through questioning and always required explanations and justifications. The large proportion of dialog related to Construction shows that expert teachers were adept at advancing their teaching based on students’ thoughts/views. Such an observation was also reported by Even et al. (1993), who found that expert teachers could flexibly use students’ responses to carry on their teaching rather than follow a fixed procedure.

Dialog related to Analysis, e.g., extracting information from tasks, logical reasoning, analyzing and solving problems, explaining, arguing, and evaluating, ranked third. The teacher often guided students, helping them analyze the problem in depth and make a breakthrough to solve it, thereby improving their ability to analyze and solve problems, at the same time, in the process of argument and evaluation, students’ logical reasoning ability. By giving examples and explanations, students developed the ability to transform complex and abstract mathematical knowledge into concrete, easily understandable knowledge and elaborate on it in their own words.

Personal Information code and its four sub-codes well reflected the ideas advocated by the current curriculum reform, which calls for connecting learning content to students’ daily lives (Personal experience), encouraging students to freely express their ideas (Subjective opinion), encouraging divergent thinking (Imagination), and developing positive attitudes and values (Emotion and value). We can see that dialog related to personal information was considerable and consisted mainly of the sub-codes, Subjective opinion (13.1% out of 16.7%) and Personal experience (2.8% out of 16.7%). The expert teachers often encouraged their students to use their imagination and express their personal opinions, thereby developing the students’ imagination, curiosity, and ability to ask questions.

The four other codes—Coordination, Speculation, Agreement and Challenge, and Guide and Instruction—occurred far less often. Several sub-codes reflecting the ideas of curriculum reform, such as Summarization (4%), Prediction and hypothesis (2.4%), Agree (3.3%), and Guide (3.4%), accounted for a relatively higher proportion, indicating that expert teachers consciously implemented curriculum reform ideas in their classrooms. However, some sub-codes important for developing students’ higher-order thinking—such as Connection (1.3%), Modeling (0.4%), Migrating application (0.3%), and Challenge (1.5%)—comprised a very low proportion of classroom dialog, suggesting that Chinese teachers (even expert ones) paid insufficient attention to developing students’ higher-order thinking in their classroom teaching.

### Comparison of classroom dialog between expert and novice teachers’ classrooms

**Figure 3:**
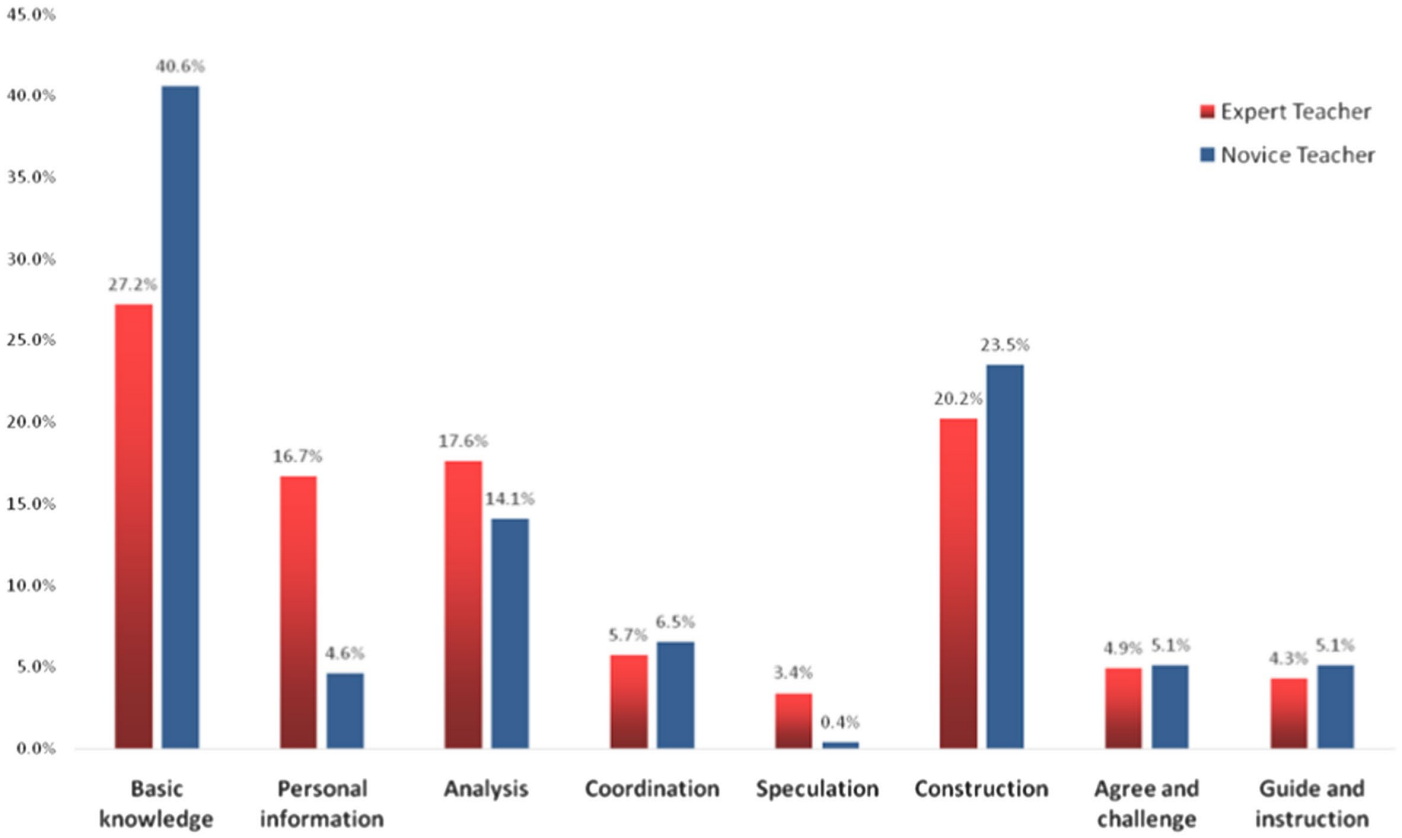
Distribution of dialogs types between expert and novice teachers’ classrooms.

Figure 3 and Table 1 show that novice and expert teachers’ classrooms shared some characteristics. For instance, both emphasized Basic Knowledge, Construction, and Analysis. Results also showed that Speculation, which signifies high-order thinking, was comparatively rare in expert and novice teachers’ classrooms.

**Table 1:** *T*-test regarding the average percentages of each code between expert and novice teachers.

Codes	Sub-codes	Expert teachers (%)	Novice teacher (%)s	*T* statistic	*P* value
Basic knowledge^*^		27.2	40.6	4.784	0.000
	Prior-known knowledge	5.4	7.8	−1.482	0.143
	Newly learned knowledge^*^	16.0	26.5	−3.503	0.000
	Repetition	5.8	6.4	−0.545	0.588
Personal Information^*^		16.7	4.6	−7.116	0.000
	Personal Experience	2.8	0.8	2.699	0.01
	Subjective opinion^*^	13.1	3.6	7.386	0.000
	Imagination	0.2	0.1	0.387	0.700
	Emotion and value	0.7	0.1	2.939	0.005
Analysis		17.6	14.1	−1.657	0.102
	Task analysis	2.6	1.9	0.904	0.369
	Exemplification	0.9	0.7	0.427	0.671
	Justification	13.2	10.7	1.256	0.231
	Evaluate	0.9	0.7	0.461	0.646
Coordination		5.7	6.5	0.767	0.445
	Connection	1.3	2.3	−2.497	0.015
	Summarization	4.0	4.2	−0.301	0.764
	Modeling	0.4	0.0	2.428	0.02
Speculation^*^		3.4	0.4	−4.081	0.000
	Prediction and hypothesis^*^	2.4	0.4	3.517	0.000
	Migrating applications	0.3	0.0	1.841	0.077
	Innovation and creation	0.7	0.0	3.079	0.004
Construction		20.2	23.5	1.321	0.191
	Probing	15.6	19.7	−2.255	0.027
	Extending	4.6	3.8	1.062	0.292
Agree and challenge		4.9	5.1	0.309	0.758
	Agree	3.3	3.6	−0.495	0.622
	Challenge	1.5	1.5	0.220	0.827
Guide and instruction		4.3	5.1	0.763	0.448
	Instruction	0.9	1.8	−1.339	0.185
	Guide	3.4	3.3	0.132	0.895

* Significant difference was found (*p* < 0.001).

However, compared with novice teachers, expert teachers had (1) a significantly lower proportion of dialog related to Basic Knowledge (27.2% vs. 40.6%, *p* = 0.000) and (2) a significantly higher proportion of dialog related to Personal Information (16.7% vs. 4.6%, *p* = 0.000) and Speculation (3.4% vs. 0.4%, *p* = 0.000).

The differences in dialog related to Basic Knowledge mainly lay in the sub-code “Newly learned knowledge.” We can see that 40.6% of classroom dialog in novice teachers’ classrooms was about basic knowledge (concepts, theorems, formulas, and operations) that was mostly new to students (26.5% out of 40.6%). As discussed earlier in the above section, expert teachers often introduced new knowledge *via* a fundamental review of prior-known knowledge, while novice teachers tended to teach new knowledge directly.

Regarding the Personal Information code, the differences mainly lay in the Subjective norm code (13.1% vs. 3.6%). Expert teachers’ classrooms also featured slightly more dialog related to Personal experience (2.8% vs. 0.8%). A closer examination of the lesson transcripts revealed that expert teachers often started their lessons with a contextual problem closely related to students’ personal experiences and asked more open questions, encouraging students to express their thoughts freely. Expert teachers tended to guide students to realize their mistakes, correct their answers, or invite other students to comment, whereas novice teachers often corrected the answer themselves. Connecting the learning content to students’ daily lives and encouraging students to express their ideas are the key ideas of the current curriculum reform. Thus, the results showed that expert teachers’ classrooms were generally more aligned with curriculum reform requirements.

Dialog related to Speculation refers to using existing knowledge and information to explore the unknown, inference, problem-solving, make hypotheses, predict the direction of things based on evidence, etc. It is a code signifying a high-order level of thinking. However, even though expert teachers had a significantly higher proportion of dialog on Speculation than novice teachers, both were quite low. It must be noted that the 33 lessons by novice teachers included no dialog on Innovation and Creation, which refers to ideas, thoughts, and opinions that are different from the norm or others. From the videotapes, we observed that novice teachers tended to adhere to the lesson plan rather than deviate based on students’ reactions. They provided students fewer opportunities to express their thoughts to ensure the lesson ran smoothly.

## Discussion and conclusion

### Discussion

This study’s results showed that expert and novice teachers paid attention to classroom dialog related to Basic Knowledge, Construction, and Analysis, the bases of Chinese mathematics lessons (Fan et al., 2015). Expert teachers facilitated significantly less dialog related to Basic Knowledge and significantly more dialog related to Personal Information and Speculation.

Based on the expert teachers’ classrooms, we can say that characteristics of effective classroom dialog in the Chinese context include ensuring students’ learning of basic knowledge, advancing students’ responses through probing or extending questions, always requiring an explanation or justification, relating the lesson content to students’ daily experiences, encouraging students to share their opinions, and facilitating inquiry-based activities.

Some of the characteristics mentioned above have been documented in the literature. For instance, that effective classroom dialog should be reciprocal and supportive (Alexander, 2008), emphasize open questions, elaboration, reasoning, and making connections (Howe et al., 2019), and make good use of students’ information to carry on their teaching rather than follow a fixed procedure (Borko and Livingston, 1989). Questioning and guidance in effective classroom dialog were more effective, strategic, and adept at constructing cognitive networks (Song et al., 2021a).

The findings reflected some unique characteristics of Chinese classrooms, such as the high proportion of dialog related to basic knowledge, justification, and probing. In China, helping students develop a profound foundation of basic knowledge and skills are important instructional goals (Ministry of Education, 2022). Requiring rigorous justification and proof is also an important characteristic of Chinese mathematics classrooms (Huang and Wong, 2007). This study shows that expert and novice teachers frequently used “probing,” an important and effective instructional strategy in China (Fan et al., 2021). The results also showed that expert teachers’ classrooms were more aligned with curriculum reform requirements, with considerable dialog related to personal information, prediction, and hypothesis, whereas novice teachers should loosen their control over the classroom and encourage more student participation.

The results also reveal that some codes related to higher-order thinking, like Evaluation, Connection, Migrating applications, Innovation, Creation, and Extending, only accounted for a low proportion of classroom dialog. It is suggested that both expert and novice teachers in China should pay attention to developing students’ high-order thinking in classrooms.

### Conclusion

This study finds that the dominant types of dialog in expert teachers’ classrooms were Basic Knowledge, Construction, Analysis, and Personal Information. Compared to novice teachers, expert teachers’ classrooms had a significantly lower proportion of dialog related to Basic Knowledge and significantly higher proportions of dialog related to Personal Information and Speculation. Expert teachers’ classrooms to some extent reflected the characteristics of effective classroom dialog in the Chinese context, which emphasize basic knowledge, probing and extending students’ responses, explanation, justification, real-life context, personal opinions, and inquiry-based activities. These characteristics not only reflect the traditional characteristics of Chinese classrooms but also the requirements of China’s current curriculum reform.

Theoretically, this study broadens our understanding of effective classroom dialog from a Chinese perspective, which may have implications for improving the quality of classroom dialog in other contexts. The framework for analyzing classroom dialog in mathematics classrooms has good reliability and validity and can be adopted in future studies.

Practically, this study can help educators in China and other educational contexts to reflect on and improve their classroom practice. It is suggested that both expert and novice teachers in China should pay attention to cultivating students’ higher-order thinking abilities through classroom dialog related to connection, modeling, migrating application and creation, etc. Novice teachers should reduce their control over the class, provide more opportunities for students to express their ideas, and increase students’ actively participate in classroom activities. For teachers in other contexts, the Chinese expert teachers’ experiences suggested that enhancing students’ learning of basic knowledge, probing and extending students’ answers through guiding questions, providing more opportunities for students to express their thoughts but always requiring a justification, facilitating inquiry-based activities, as well as flexibly adjust the instruction based on students’ reactions, are effective strategies to increase the effectiveness of classroom dialog.

This study has several limitations. First, as its analysis was based on lesson transcripts, this study only examined the frequencies of different types of classroom dialog. Future studies could extend the analysis to include the duration of each code to yield more comprehensive results. Second, this study did not analyze teachers’ and students’ talk separately. As the current curriculum reforms call for students to play a central role in class, it would be worth examining students’ different responses. Third, due to the relatively large number of teachers, only one lesson was selected from each teacher. Last, only quantitative data were reported. A qualitative analysis of classroom transcripts could enrich the results. These limitations will be considered in our subsequent studies.

## Data availability statement

The raw data supporting the conclusions of this article will be made available by the authors, without undue reservation.

## Ethics statement

The studies involving human participants were reviewed and approved by Beijing Normal University. The patients/participants provided their written informed consent to participate in this study.

## Author contributions

YC, WZ, and JM: conceptualization. WZ and JM: methodology, formal analysis, and writing-original draft preparation. YC and WZ: data collection, writing-review, and editing. YC: supervision and project administration. All authors contributed to the article and approved the submitted version.

## Funding

This study was fund by the China National Education Sciences Grant (2022): Evaluating classroom instruction through key classroom activities with the support of Artificial Intelligence (Grant No. CHA220300) and Faculty of Education, Beijing Normal University.

## Conflict of interest

The authors declare that the research was conducted in the absence of any commercial or financial relationships that could be construed as a potential conflict of interest.

## Publisher’s note

All claims expressed in this article are solely those of the authors and do not necessarily represent those of their affiliated organizations, or those of the publisher, the editors and the reviewers. Any product that may be evaluated in this article, or claim that may be made by its manufacturer, is not guaranteed or endorsed by the publisher.

## Footnotes

^1^ https://159.75.97.242/
