# Supplementary material for: What is effective classroom dialog? A comparative study of classroom dialog in Chinese expert and novice mathematics teachers’ classrooms
Source: Front Psychol. 2022 Sep 23;13:964967. doi: 10.3389/fpsyg.2022.964967 (PMC9537045; doi:10.3389/fpsyg.2022.964967)
Supplement: Supplementary file 1 [file Data_Sheet_1.docx]

# Appendix1

The analytical coding framework for classroom dialogue

| **Codes** | **Sub-codes** | **Descriptions** |
| --- | --- | --- |
| **(Invite or response)**  **Basic knowledge** | **Prior-known knowledge** | Review the knowledge of concepts and symbols, relational operations, the history of mathematics, general knowledge, and other knowledge learned before the lesson. |
|  | **Newly learned knowledge** | Present the knowledge on concepts and symbols, relational operations, the history of mathematics, general knowledge, and other knowledge to be learned in the lesson. |
|  | **Repetition** | Repeating the words of others. |
| **(Invite or response)**  **Personal information** | **Personal experience** | Sharing personal experience, direct activity experience, etc. |
|  | **Subjective opinion** | Freely express ideas and opinions about something with no need to provide rigorous justification. |
|  | **Imagination** | Come up with wild ideas or ask questions through imagination |
|  | **Emotion and value** | Expresses curiosity, interest, or desire to learn about new issues, problems, and questions. |
| **(Invite or response)**  **Analysis** | **Task analysis** | To break down a question, extract information from it, or find a breakthrough in solving it. |
|  | **Exemplification** | To understand particular knowledge by giving concrete examples or counter-examples. |
|  | **Justification** | To provide logical reasoning, explanations, and arguments about a topic or problem. |
|  | **Evaluate** | To evaluate the opinions of others with analysis and reasons |
| **(Invite or response)**  **Coordination** | **Connection** | Finding logical connections between mathematical knowledge and grasping the structure and relevance of knowledge as a whole. |
|  | **Summarization** | Making comparing and summarizing patterns based on the connections and similarities between knowledge. |
|  | **Modeling** | The process of formulating practical application problems in mathematical notation and language and building mathematical models to solve them. |
| **(Invite or response)**  **Speculation** | **Speculation** | Using existing knowledge and information to explore the unknown, make inferences, speculate on conclusions, and predict the direction of results based on evidence. |
|  | **Migrating Applications** | The transfer and application of mathematical knowledge and skills, as well as mathematical thinking methods in another context. |
|  | **Innovation and creation** | To propose different ideas, thoughts, opinions, etc. based on existing ideas or thoughts. |
| **(Invite or response)**  **Construction** | **Probing** | Clarifying questions, probing questions, and further discussion. |
|  | **Extending** | Extending mathematical knowledge, exploring different ideas and approaches to a problem, or integrating interdisciplinary knowledge to develop students’ thinking. |
| **Agree and Challenge** | **Agree** | Agree or accept an answer |
|  | **Challenge** | Challenge/disagree/ unaccepted/ doubt an answer |
| **Guide and Instruction** | **Guide** | Provide assistance and support according to the student’s learning pace and cognitive level. |
|  | **Instruction** | The teacher gives clear instructions on how to organize learning activities and asks others to respond accordingly. |

# Appendix 2

An example of the coding results

|  | Role | Content | Initial or Response | Categories | Sub-categories |
| --- | --- | --- | --- | --- | --- |
| 1 | T | Before the lesson starts, I want to ask, what is inequality in one unknown? Cake. | I | Basic knowledge | Prior-knowledge |
| 2 | S | There is only one unknown, and the power of the letters is one. | R | Basic knowledge | Prior-knowledge |
| 3 | T | Okay, the power of the unknown is one, the inequality is called inequality in one unknown; the next question is, which step is the most important in order to solve the inequality in one unknown? | I | Basic knowledge | Prior-knowledge |
| 4 | S | The symbol of inequality. | R | Basic knowledge | Prior-knowledge |
| 5 | T | The symbol of inequality, do you all agree on this? | I | Agree and Challenge | Challenge |
| 6 | S | The last step | R | Basic knowledge | Prior-knowledge |
| 7 | T | Which step? | I | Construction | Probing |
| 8 | S | If both sides are multiplied with a negative number, the inequality sign has to be changed. | R | Basic knowledge | Prior-knowledge |
| 9 | T | The inequality sign has to be changed, it may not be the last step, just that we have to multiply or divide a negative number with both sides, the direction of the unequal sign has to be changed, this is an important step.  Today we will talk about [Writing on the board] the ways to solve inequalities in one unknown. Turn your book to page forty-two, page sixty-two! |  | Agree/Challenge  Basic knowledge | Challenge  Present new knowledge |
| [Student working on question][Teacher writing on the board][Teacher walking around] | | |  |  |  |
| 10 | T | Check your answers when you’ve finished. |  | Guide and Instruction | Instruction |
| 11 | T | Okay, put down your pens. Most of you have finished already; answer the first question, x minus five is smaller than a negative one. Raise your hand before you answer. | I | Basic knowledge | Present new knowledge |
| 12 | S | x is smaller than four. | R | Basic knowledge | Present new knowledge |
| 13 | T | x is smaller than four. In the second question, four x is larger than twelve. | I | Basic knowledge | Present new knowledge |
| 14 | S | x is larger than three. | R | Basic knowledge | Present new knowledge |
| 15 | T | x is larger than three; are there any other answers? | I | Construction | Extending |
| 16 | S | x is larger than negative three. | R | Construction | Extending |
| 17 | T | x is larger than negative three; tell her why you think it is x larger than negative three. | I | Analysis | Justification |
| 18 | S | Because both sides are divided by negative three, nine divided by negative three… | R | Analysis | Justification |
| 19 | T | Oh, that’s right, nine divided by negative three gives you negative three, and the unequal sign has to be changed.  Both sides of the inequality have to be multiplied with a negative number, and the direction of the unequal sign has to be changed, nine divided by negative three; what is the result then? Negative three, okay.  The next question, how would you do it? | I | Agree/Challenge  Analysis | Accept  Justification |
| 20 | S | Move x to the left-hand side first, it equals negative x. Move eight to the right, it equals negative eight. | R | Analysis | Justification |
| 21 | T | Okay, everyone, eight is larger than x, move x to the left-hand side; we have to change the symbol, so we move eight to the right-hand side, and the direction of the unequal sign is not changed. What can we do then? | I | Construction | Probing |
| 22 | S | Divide both sides with a negative one. | R | Construction | Probing |
| 23 | T | x is smaller than eight, the answer is right. Are there any other ways to deal with it? | I | Construction | Extending |
| 24 | S | The other way is to move the items first, so negative x is larger than negative eight, and multiply both sides of the inequality with a negative one, so we get x is smaller than eight. | R | Construction | Extending |
